# Enhancing multi-UAV air combat decision making via hierarchical reinforcement learning

**DOI:** 10.1038/s41598-024-54938-5

**Published:** 2024-02-23

**Authors:** Huan Wang, Jintao Wang

**Affiliations:** 1 College of Artificial Intelligence and Automation, Hohai University, Changzhou, 213200 China; 2 College of information and Network Engineering, Anhui Science and Technology University, Chuzhou, 233030 China; 3 School of Electrical and Information Engineering, Wanjiang University of Technology, Maanshan, 243000 China

**Keywords:** Aerospace engineering, Information theory and computation, Computer science, Information technology, Software, Aerospace engineering, Information theory and computation, Computer science, Information technology, Software

## Abstract

In the realm of air combat, autonomous decision-making in regard to Unmanned Aerial Vehicle (UAV) has emerged as a critical force. However, prevailing autonomous decision-making algorithms in this domain predominantly rely on rule-based methods, proving challenging to design and implement optimal solutions in complex multi-UAV combat environments. This paper proposes a novel approach to multi-UAV air combat decision-making utilizing hierarchical reinforcement learning. First, a hierarchical decision-making network is designed based on tactical action types to streamline the complexity of the maneuver decision-making space. Second, the high-quality combat experience gained from training is decomposed, with the aim of augmenting the quantity of valuable experiences and alleviating the intricacies of strategy learning. Finally, the performance of the algorithm is validated using the advanced UAV simulation platform JSBSim. Through comparisons with various baseline algorithms, our experiments demonstrate the superior performance of the proposed method in both even and disadvantaged air combat environments.

## Introduction

UAV have found widespread applications in diverse military domains such as surveillance, reconnaissance, and operations due to their agility, cost-effectiveness, and reduced risk of casualties. In recent years, drones have significantly influenced numerous military operations worldwide. Notably, substantial scientific efforts worldwide have been invested in UAV air combat decision-making technology research. For instance, in the United States, Psibernetix developed the “Alpha” AI, an artificial intelligence system that leverages genetic algorithms for air combat decision-making. This system triumphed over a retired United States Air Force colonel in simulated air combat scenarios^1^. Concurrently, the U.S. Defense Advanced Research Projects Agency (DARPA) initiated the Offensive Swarm Tactics (OFFSET) project, focusing on developing diverse swarm tactics for unmanned aircraft to support small ground forces in complex urban settings^2^. Additionally, the multinational Future Combat Air System (FCAS) project in Europe aims to bolster human-machine interaction, garnering participation from multiple nations. Russia’s Sukhoi Design Bureau has spearheaded the S-70 “Hunter” UAV and embarked on research on technology for cooperation between this UAV and the Su-57 fighter aircraft. Furthermore, researchers worldwide have explored various UAV air combat decision-making methodologies. McGrew et al.^3^ introduced dynamic programming to enhance efficiency in 1v1 air combat game decision-making, while Wu et al.^4^ proposed a fuzzy rule-based decision-making method that guides fighters to execute more tactically advantageous moves. However, the majority of these approaches rely on rule-based design, which faces challenges in solving complexity and designing rules for multi-UAV air combat environments.

Deep reinforcement Learning (DRL) techniques have gradually found applications in air combat decision-making. DRL, a subset of machine learning methods, possesses strong adaptability and autonomous learning capabilities, without necessitating extensive professional background knowledge. we propose a hierarchical decision-making approach for multi-UAV air combat scenarios to improve the training efficiency. Our main contributions are as follows:

- A hierarchical decision network is designed to reduce the spatial dimensions of action decision making.
- we propose an empirical decomposition mechanism to break down complex task experiences for enhancing training efficiency.
- The performance of our algorithm under different JSBSIM simulation environments is evaluated.

The subsequent sections of this paper are organized as follows: Section "Related work" provides a review of related work in the field. Section "Method" presents the framework of our method, delineating the hierarchical decision-making network and the experience decomposition mechanism tailored for the multi-UAV air combat environment. The experimental results and validation of the algorithm are detailed in Section "Experiment". Lastly, Section "Conclusion" presents the conclusions drawn from this study.

## Related work

This paper primarily focuses on integrating hierarchical reinforcement learning techniques with the unique characteristics of the multi-UAV air combat environment to enhance decision-making efficiency. Currently, numerous scholars have conducted research on air combat decision-making. This section initially provide an overview of existing techniques in UAV air combat decision-making. Subsequently, it introduces relevant hierarchical reinforcement learning techniques.

### Air combat decision-making techniques

Air combat decision-making stands as the pivotal challenge within the domain of UAV air combat games. Presently, decision-making theory rooted in the OODA(Observation, Orientation, Decision, Action) loop serves as a widely adopted framework. UAV formulate tactical decisions by assimilating information obtained from the battlefield environment, amalgamating it with their own state to generate strategic maneuvers. Scholars across various nations have proposed numerous method to address the air combat decision-making problem and these methods can be categorized based on their solution approaches into methods grounded in countermeasure theory, those based on expert systems, and heuristic learning methods.

The first category revolves around countermeasure theory. For instance, Wayne et al.^5^ introduced a “two target game” model that delineates both sides of an air battle, where each side can act as either the pursuing or fleeing party depending on the prevailing battlefield situation. Austin et al.^6^ proposed a matrix-based approach for maneuver decision-making. This methodology involves discretizing maneuvers into a maneuver library that comprises various basic maneuvers. Subsequently, it depicts all potential maneuver combinations between opposing sides using matrices. Finally, it solves the aircraft’s equation of motion through numerical integration to derive the optimal decision sequence.

The second category involves the expert system approach. For instance, Xi et al.^7^ incorporated the power potential field into the decision-making process for multi-UAV air combat, thereby enhancing cooperative performance in such scenarios. Zhou et al.^8^ developed a multi-UAV autonomous control algorithm based on the ant colony algorithm to enhance the success rate of UAV clusters in aerial combat. Yan et al.^9^ combined the collaborative particle swarm algorithm with collaborative functions and variables to address the constraint problem associated with simultaneous attacks by multiple UAV.

The third category comprises heuristic learning methods, with reinforcement learning being the most prevalent approach. This method emulates the neuron structure of the human brain in network topology, enabling the characterization of nonlinear and intricate relationships alongside corresponding learning capabilities. For instance, Zhou et al.^10^ proposed an improved method of situation assessment for the air combat environment. Sun et al.^11^ introduced a multi-intelligence hierarchical strategy gradient algorithm, achieving tactical strategies that surpass human expert cognition through self-game training. Shi et al.^12^ developed a proximal strategy optimization algorithm tailored for air combat decision-making, thereby enhancing the decision-making performance of fighters in 3v3 air combat scenarios. Additionally, bionics-based algorithms^13^ find frequent application in the realm of air combat decision-making.

The three types of above methods have the following problems.

- Countermeasure theory-based methods pose challenges in solving and constructing models, and they are more suitable for simpler air combat scenarios such as like pursuit and interception.
- Methods grounded in expert systems heavily rely on the expertise and decision-making proficiency of specialists, demanding a high level of professional background from designers. However, when the air combat environment becomes intricate and variable, relying solely on expert knowledge may hinder optimal decision-making.
- Research based on heuristic learning methods often focuses on simpler scenarios such as pursuit, interception, and one-on-one air combat, which may limit their applicability in complex air combat scenarios. While some studies have been extended to combat scenarios involving multiple aircraft, the adaptation to such intricate environments remains a challenge.

While certain studies have ventured into the realm of multi-UAV air combat, achieving desired training outcomes often proves challenging and may fall short of expectations. We propose an algorithm for multi UAV combat environments, which does not rely on expert experience and uses empirical decomposition mechanism to improve the experience quality of the experience. In addition, we design a hierarchical decision network to solve the problem of large action dimension space and difficult decision-making of agents in complex UAV combat environments.

### Hierarchical reinforcement learning

Hierarchical reinforcement learning stands as a significant branch within the field of reinforcement learning, drawing inspiration from the concept of solving complex problems by breaking them down into several subproblems. This approach involves decomposing a complex problem into smaller, more manageable subtasks, thereby solving them sequentially through task decomposition to address the overarching complex issue. Current task decomposition methods can be broadly categorized into two groups: (1) all subproblems collaborate to complete the decomposed task collectively; (2) the outcome of the prior subproblem serves as input for solving the subsequent subproblem, resolving the problem in a hierarchical manner^14–16^. For instance, Wang et al.^2^ employed hierarchical reinforcement learning to tackle the mobile robot navigation problem, effectively addressing the limitations of conventional navigation methods in complex environments. Similarly, Yang et al.^17^ applied a hierarchical network multiagent learning framework to enhance decision-making in 3v3 football match environments, thereby raising the multiagent gaming confrontation level. Moreover, hierarchical reinforcement learning has found applications in diverse scenarios such as robotic arm control, StarCraft II gaming, and various other domains^18,19^.

## Method

In this paper, we present a hierarchical reinforcement learning-based method for multi-UAV air combat decision-making. This section focuses on outlining the design of our hierarchical decision-making approach for multi-UAV air combat scenarios. Initially, we model the multi-UAV air combat scenario using a partially observable Markov process. Subsequently, we introduce a network framework tailored for hierarchical decision-making in multi-UAV air combat algorithms, leveraging hierarchical reinforcement learning technology to streamline the complexity involved in maneuver decision-making. Finally, we propose a decomposition transformation mechanism that aims to break down high-quality adversarial experiences. It also to augment the presence of high-quality adversarial experiences within the experience buffer, consequently enhancing the efficiency of strategy learning.

### Decentralized partially observable markov decision processes (DEC-POMDP)

In a multi-UAV air combat scenario, the UAV agent lack access to global information. This paper considers the multi-UAV air combat decision-making task as a DEC-POMDP, characterized by the tuple $\left\langle S, A, P, r, Z, O, n, \gamma \right\rangle$. Here, $s\in S$ denotes the current state of the environment. At each time step, an agent $g\in G\equiv \left\{ 1,....,n \right\}$ selects an action $a^{g}\in A$, collectively forming a joint action $a\in A\equiv A^{g}$, leading to changes in the environment via the state transition function $P\left( s^{'}|s,a\right) :S\times A\times S\rightarrow [0,1]$ governing state transitions. Throughout this process, all agents share a common reward function $r\left( s,a\right) :S\times A \rightarrow {\mathbb {R}}$, with $\gamma \in [0,1)$ representing the discount factor.

In a partially observable environment, each agent receives an individual observation state denoted as $o \in Z$ via the state transfer function $O\left( s,a\right) :S\times A \rightarrow Z$. Additionally, the action observation history for each agent can be represented as $\tau ^{g}\in T\equiv \left( Z\times A\right) ^{*}$, while the random strategy $\Omega ^{g}\left( a^{g}|\tau ^{g}\right) :T\times A\rightarrow [0,1]$ serves as a condition. The joint action-value function for the joint strategy $\Omega$ can be formulated as shown in Eq. (1).

1 $$\begin{aligned} Q^{\pi }\left( s_{t},a_{t} \right) = E_{s_{t+1:\infty } ,a_{t+1:\infty }}[R_{t}|s_{t},a_{t}], \end{aligned}$$

where, $R_{t}={\sum }^{\infty }_{i=0}\gamma ^{i}r_{t+i}$ represents the discount reward. The strategy learning of each agent in training takes only its own action observation history as input.

### Overall structure of the proposed algorithm

**Figure 1:**
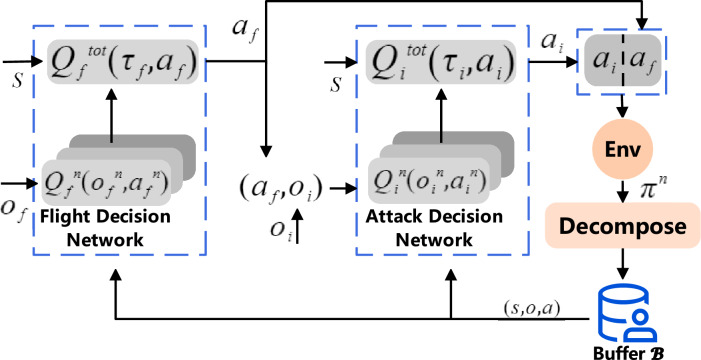
Framework of the hierarchical decision-making multi-UAV air combat method.

The network framework of the hierarchical decision-making method for multi-aircraft air combat, designed in this paper, is illustrated in Fig. 1. The framework primarily consists of the experience buffer, flight action decision-making layer, attack action decision-making layer, environment interaction segment, and confrontation experience decomposition and transformation segment. Training commences by extracting data samples from the experience buffer ${\mathscr {B}}$ to facilitate the training and updating of the two decision-making layers. Subsequently, the resulting flight action $a_f$ and attack action $a_i$ combine to form a set of actions that is inputted into the environment interaction segment. This segment updates the environment state based on the input action combination and generates single-step combat experiences that are deposited into $\pi ^n$ and concurrently employed for flight and attack decision-making. Simultaneously, the segment produces observation states $o_f$ and $o_i$, which are utilized in flight and attack action decision-making, as delineated in Eqs. (2) and (3).

2 $$\begin{aligned}{} & {} o_f = \{o^1,...,o^n\}, \end{aligned}$$

3 $$\begin{aligned}{} & {} o_i = {\omega ^m\odot o_f}, \end{aligned}$$

where $\odot$ denotes the elementwise product of the two arrays and *N* represents the total number of agents. The coefficient series $\omega ^m = ({ \omega ^{m,1},... \omega ^{m,n},... \omega ^{m,N} })$ corresponds to the current observation state ($o^1,...,o^n$) of each agent. When the distance between agent *m* and *n* falls below the attack range threshold, $\omega ^{m,n} = 1$. Conversely, if the distance exceeds this threshold, $\omega ^{m,n} = 0$.

The experience decomposition segment decomposes the generated experience $\pi ^n$ and stores it in the experience buffer ${\mathscr {B}}$. The content within the experience buffer gradually undergoes replacement based on the degree of novelty once the buffer reaches full capacity, iteratively continuing until the training concludes.

The flight action and attack decision layers are designed based on the QMIX^20^ value decomposition network. The flight action decision layer trains the joint action value function $Q_f^{\mathrm{{tot}}}\left( {\tau }_f,a_f \right)$ of the centralized flight action layer, while the attack action decision layer trains the joint action value function $Q_i^{\mathrm{{tot}}}\left( {\tau }_i,a_i \right)$ specific to the attack action layer. Both action value functions can be expressed as the sum of individual intelligence value functions $Q_g(\tau ^{j},a^{j},\theta ^{j} )$, as detailed in Eq. (4).

4 $$\begin{aligned} Q^{\textrm{tot}}(\tau ,a)=\sum _{j=1}^{n} Q_j(\tau ^{j},a^{j},\theta ^{j}), \end{aligned}$$

It is also necessary to ensure that $Q^{\mathrm{{tot}}}$ satisfies Eq. (5).

5 $$\begin{aligned}{} & {} {\mathop {\arg \max }_{a}Q^{\textrm{tot}}(\tau , a )=\left( \begin{matrix} {\mathop {\arg \max }}_{a^{1}}Q_1(\tau ^{1}, a^{1}) \\ {...}\\ {\mathop {\arg \max }}_{a^{n}}Q_n(\tau ^{n}, a^{n}) \end{matrix}\right) ,} \end{aligned}$$

6 $$\begin{aligned}{} & {} \frac{\partial Q_{\textrm{tot}}}{\partial Q_g} \geqslant 0,\forall g\in G, \end{aligned}$$

We transform Eq. (5) to a monotonicity constraint as in Eq. (6), and implement it using a hybrid network. The loss function is given in Eq. (7).

7 $$\begin{aligned}{} & {} {\mathscr {L}}\left( \theta \right) =\sum ^{b}_{i=1}\left[ \left( y^{\textrm{tot}}_{i}-Q^{\textrm{tot}}\left( \tau ,a,s;\theta \right) \right) ^2\right] ,{} & {} \end{aligned}$$

where, b is the sample batch size for each training, $y^{\textrm{tot}}=r + \gamma * \mathrm max_{a^{'}} Q^{\mathrm{{tot}}}\left( \tau ^{'},a^{'},s^{'};\theta ^{-}\right)$, and $\theta ^{-}$ represents the target network parameters . $Q_f^n\left( {o_f}^n,{a_f}^n \right)$ and $Q_i^n\left( {o_i}^n,{a_i}^n \right)$ represent the action value function of each agent for generating flying and attacking actions, which are calculated according to Eq. (1).

### Hierarchical decision-making network

Multi-UAV combat poses a complex challenge, necessitating optimal attack decision-making amid rapidly evolving battlefield conditions. This paper proposes to segregate the UAV decision control task into distinct flight actions $a_f$ and attack actions $a_i$ based on their specific characteristics. The flight maneuver entails controlling the UAV’s flight angle, speed, and altitude, which requires a comprehensive assessment of the global battlefield situation while evading enemy missiles. Conversely, the attack maneuver focuses solely on choosing whether to engage in an attack and the target for the attack, considering localized battlefield information. As depicted in Fig. 1, the flight action $a_f$ is generated by the flight decision layer, which encompasses heading, altitude, and speed decisions, necessitating a comprehensive array of battlefield information ($o_f$). On the other hand, the attack action $a_i$ is produced by the attack decision layer, which determines whether to engage in an attack and the number of targets and solely considers information concerning nearby enemy aircraft and adjacent friendly forces ($o_i$). The combination of flight and attack actions orchestrates the control of UAV to accomplish their air combat missions.

### Empirical decomposition mechanisms

**Figure 2:**
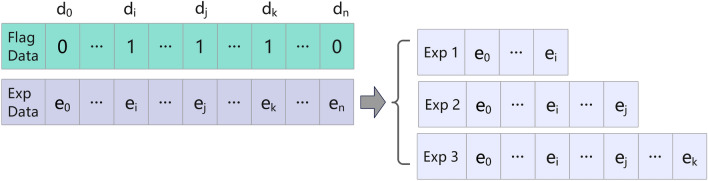
Schematic diagram of experience decomposition mechanisms.

Winning in a multi-UAV game demands continuous, efficient maneuver decision-making by agents over extended periods. Traditional methods that directly train agents to accomplish complex tasks encounter two primary issues: (1) limited experience availability at the onset of training, leading to a scarcity of high-quality experience; (2) lengthy sequences of adversarial rounds resulting in inefficient direct learning training. To address these challenges, a prior study^21^ introduced the hindsight experience replay method, which utilizies random point decomposition for sampled data; however, the method neglects to consider the specific stage characteristics of task experience. In this paper, we propose an enhanced experience decomposition technique, aiming to increase the initial training experiences while simultaneously reducing training complexity. Inspired by humans’ learning of complex tasks, our method decomposes intricate tasks into distinct learning stages, as depicted in Fig. 2. Initially, for each round of confrontation experience data, synchronized flag data point *d* is recorded. Bits set to 1 (e.g., $d_i$,$d_j$,$d_k$) correspond to instances of downing enemy fighters, with the remaining bits being set to 0. Subsequently, based on these flag data, bits set to 1 represent moments of enemy fighters being downed. The subsequent step involves decomposing the data into experiences 1, 2, and 3, contingent upon the position of the 1 bit in the flag data (e.g., $e_i$, $e_j$, $e_k$), and recalculating the associated rewards. Ultimately, both the original experience data and the decomposed experience data are stored in the experience buffer ${\mathscr {B}}$ for model training. This method not only expands the experience gained during each air combat round but also breaks down the complex battle process into several stages (destroying different numbers of fighters), thereby alleviating the learning complexity of the model in strategy adaptation.

In our method, both the flight decision network and the attack decision network utilize data from the experience buffer combined with Eq. (7) for updating and selecting their respective actions. These actions interact with the environment to generate new confrontation experiences through the $\epsilon -$greedy algorithm, as illustrated in Eq. (8). The round experience $\pi ^n$ derived from each confrontation round undergoes decomposition and transformation, as detailed in Eq. (9), to yield a set $\pi _{\textrm{sub}}^n$ comprising subtarget experience data that are utilized for updating the experience buffer. In this formula, $\pi _j$ represents the experience set for the first *j* steps of the round experience $\pi ^n$, while $d_j$ signifies the flag data bit in the countermeasure data. $d_j = 1$ denotes the moment when an enemy UAV has been shot down. Details of the proposed algorithm can be found in the file **algorithm.pdf** in supplementary material.

8 $$\begin{aligned}{} & {} a=\left\{ \begin{array}{l} \textrm{argmax}_{a}Q(o,a), \varepsilon , \\ random\, action, 1 - \varepsilon , \end{array}\right\} . \end{aligned}$$

9 $$\begin{aligned}{} & {} \pi ^n \mapsto \pi _{\textrm{sub}}^n :\{\pi _j|d_j=1,j\in [0,t_{round}]\}, \end{aligned}$$

## Experiment

The experimental setup in this paper is constructed using the JSBSim simulation platform. The experimental server utilized is equipped with an Intel Xeon Silver 4210R CPU, an NVIDIA GeForce RTX 3080 graphics card, and 64GB of memory.

### Multi-UAV combat scenario design

In the devised combat scenario, our UAV encounter enemy UAV while on patrol, initiating air combat within low-altitude airspace. The objectives include destroying the enemy aircraft, eliminating all enemy UAV, or determining the winner based on the surviving side with more UAV by the end of the combat period. The experimental sampling occurs at a step of 1 second, with each round of combat lasting 600 seconds. The initial distance between the two sides is approximately 8 kilometers, and the horizontal heading is set at $0^\circ$, incrementing in a clockwise direction. Details regarding the main performance and initial state configurations are shown in Table 1.

**Table 1:** Parameter and initial state of aircraft.

Categories	Value range	Categories	Value range
Radar detection range	[0,1800m]	UAV speed range	[50,300]m/s
Number of missiles	6	Missile range	[0,1200]m
Initial speed	200m/s	Initial height	2000m
Initial course (ally)	$0^{\circ }$	Initial course (enemy)	$180^{\circ }$
Max ATA	$45^{\circ }$	Missile speed range	[0,500]m/s

The Max ATA (Antenna Train Angle) is angle between the longitudinal axis of allied UAV’s flight and the radar sight line that detects enemy UAV.

### Model building

Using the multi-UAV air combat method based on hierarchical reinforcement learning proposed in this paper to construct a reinforcement learning agent, the relevant elements involved in reinforcement learning are defined according to the method described in this paper.

- State space design
  The status information encompasses details regarding both allied and enemy UAV, as well as launched missiles. Owing to the constraints of radar detection range, certain information regarding the position, heading, or speed of enemy UAV or missiles might be unavailable. In such cases, the missing information is recorded as zero. Additionally, all the data is descaled. Please refer to Table 2 for a comprehensive breakdown of the state space information.

**Table 2:** State space information.

Entity name	State information	Entity name	State information
Ally	Position, Course, Speed, Number of missiles	Enemy	Position, Course, Speed
Ally missle	Position, Course, Speed	Enemy missle	Position, Course, Speed

The observed enemy UAV and enemy UAV missile information and the allied UAV state information mentioned in Table 2 are shared between UAVs. However, using all the shared information will reduce the training efficiency, so we propose hierarchical decision networks to share specific state information between UAVs for different action decisions. We select different state information for training based on the needs of different action decisions. For the flight action decision network, the global observable state information is shared as input so that the UAVs can decide the flight direction and avoid missiles according to the overall battlefield situation, while the attack action decision network uses the individual UAV observable state information as input to generate the optimal attack target.

- Action space design
  In this paper, a UAV’s decision-making actions encompass both flight control actions and attack actions. Flight control maneuvers involve adjustments in heading, altitude, and speed, while attack maneuvers encompass the decision of whether to initiate an attack and determining the number of enemy aircraft to engage. To diminish the spatial complexity of decision-making actions, this paper discretizes the flight control actions, as outlined in Table 3.

**Table 3:** Action space information.

Action	Value	Action	Value
Course	Turn left, Hold on, Turn Right	Height	Pull up , Hold on, Dive
Speed	Decelerated fligh, Steady flight, Accelerated flight	Attack	Not attack, Target ID

- Reward design
  The reward structure comprises two components: the detection class reward, denoted as $r_d$, and the attack class reward, denoted as $r_i$. The flight decision network undergoes training using the combined sum of $r_d$ and $r_i$, while the attack decision network is trained exclusively using the obtained attack reward, $r_i$. The specific reward design categories are shown in Table 4.

**Table 4:** Reward shaping.

Event	Reward	Event	Reward
Kill	10	Missile escaped	2
Out	− 2	Detect the enemy	3
Win	30	Lose	− 30
Draw	− 6

- Network design
  The neural network architecture devised in this paper comprises a two-layer parallel structure, specifically the flight and attack decision layer networks. Both networks share a similar structure, employing a double hidden layer network configuration. Prior to entering the network, the input states necessitate normalization. The hyperparameters governing these networks are detailed in Table 5.

**Table 5:** Neural network hyperparameters.

Parameter	Value	Parameter	Value
Optimizer	Adam	Replay buffer Size	3000
Learn rate	3e−4	Discount factor	0.96
Batch size	300	Initial expert sample size	1000

### Training result

**Figure 3:**
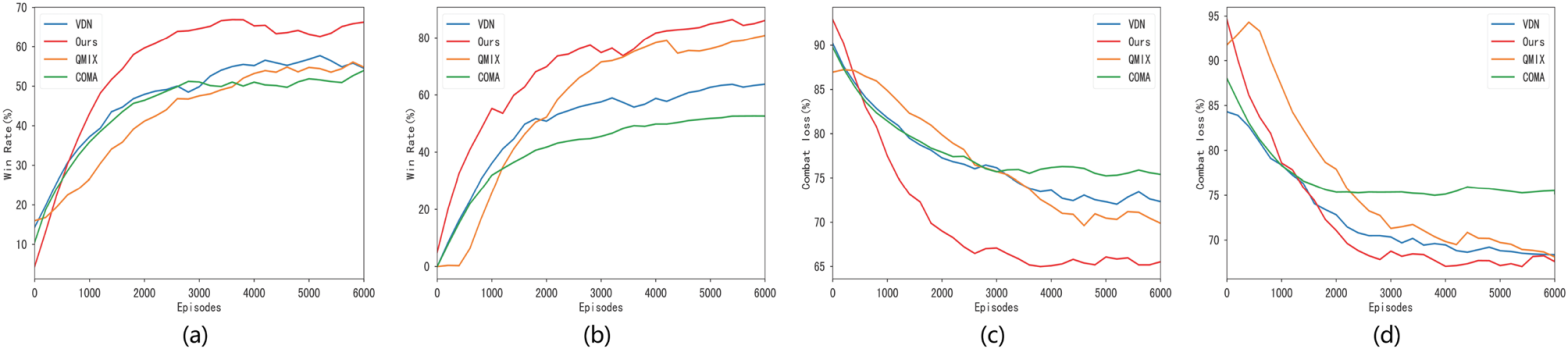
Algorithm comparison diagram. (**a**) Comparison chart of the win rate (4v4); (**b**) Comparison chart of the win rate (8v8); (**c**)Comparison chart of the combat loss rate (4v4); (**d**) Comparison chart of the combat loss rate (8v8).

To validate the efficacy of the algorithms proposed in this paper, models developed using the design methodology described above were benchmarked against several baseline algorithms in a homogeneous adversarial environment. The comparison algorithms encompassed common multi-intelligence adversarial algorithms such as the VDN algorithm, COMA algorithm^22^, and QMIX algorithm. The experiments were conducted, and win rates and combat loss rates were measured. Combat loss rate indicates the percentage of lost UAV by the conclusion of the battle. The experiments were executed through 10 rounds of training in 4v4 and 8v8 combat scenarios. The outcomes of the training and comparison illustrated represented in Fig. 3. The depicted curves represent the average outcomes across multiple training rounds. The results show that the method proposed in this paper achieves notably superior win rates and reduced combat loss rates compared to the baseline comparison algorithms. Additionally, the proposed method demonstrates faster convergence during training. Specifically, our method begins converging after 100 training rounds, while the QMIX and VDN methods converge after 300 training rounds. Although the COMA method shows a convergence rate similar to our method, its convergence outcomes are inferior.

To evaluate the ultimate performance of the algorithms, experiments were carried out in 4v4 and 8v8 battle scenarios. The final models were trained using each algorithm for confrontation testing, with testing encompassing a total of 100 rounds, and with 500 games conducted per round.Every rounds of testing use the random seeds in the range of 1-100 which generated by a random function. The average test results are presented in Table 6. Analyzing the table, it is evident that our method outperforms the comparison algorithms in terms of the win rate, the loss rate, and stability.

The experiments described above demonstrate that the method proposed in this paper outperforms other baseline comparison algorithms with regard to the confrontation win rate and combat loss rate. Moreover, the training convergence speed is rapid, and the performance exhibits relatively stable behavior. These findings collectively validate the effectiveness of the algorithm.

**Table 6:** Algorithm test results.

Algorithm	Win rate	std	Combat loss	std
Ours(4v4)	**0.647**	**0.036**	**0.695**	**0.020**
QMIX(4v4)	0.588	0.103	0.725	0.055
VDN(4v4)	0.612	0.032	0.759	0.022
COMA(4v4)	0.516	0.028	0.809	0.015
Ours(8v8)	** 0.829**	**0.041**	**0.655**	**0.011**
QMIX(8v8)	0.818	0.043	0.683	0.016
VDN(8v8)	0.730	0.034	0.690	0.014
COMA(8v8)	0.442	0.048	0.723	0.019

Significant values are in bold.

### Ablation studies

The algorithm presented in this paper enhances the QMIX algorithm in two aspects. To assess the impact of various mechanisms on the performance enhancement of the algorithm, ablation experiments are conducted. These experiments involve removing one improvement from the proposed algorithm at a time and comparing the training outcomes in 4v4 and 8v8 adversarial environments. The configurations of the three comparison algorithms are outlined in Table 7, where $\checkmark$ signifies the inclusion of the corresponding improvement mechanism, and $\times$ signifies its exclusion.

**Table 7:** Ablation experiment setting.

Algorithm	Hierarchical network	Transformation
Ours	$\checkmark$	$\checkmark$
QMIX	$\times$	$\times$
Ours-noH	$\times$	$\checkmark$
Ours-noET	$\checkmark$	$\times$

**Figure 4:**
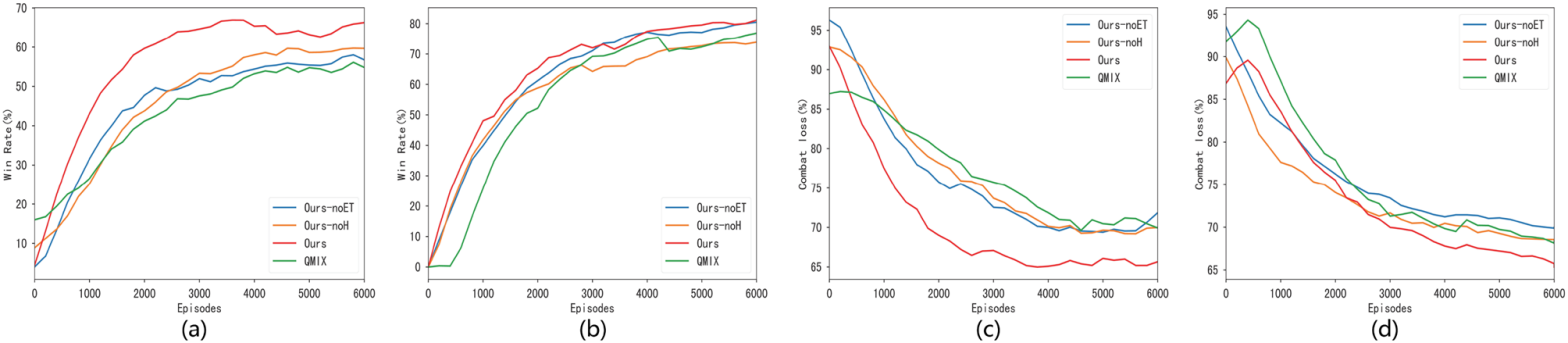
Ablation experiment results. (**a**) Comparison chart of the win rate (4v4); (**b**) Comparison chart of the win rate (8v8); (**c**) Comparison chart of the combat loss rate (4v4); (**d**) Comparison chart of the combat loss rate (8v8).

Figure 4 illustrates the comparison results of the ablation experiment algorithms. Specifically, Figure 4a and b depict the comparison of victory rates during the ablation experiment training process, while Figure 4c and d present the comparison of combat loss rates during the same training process.

The figures show that the impact of removing a specific improvement method varies. In terms of algorithm stability, both the Ours-noH and Ours-noET methods exhibit better stability than the QMIX method. Regarding convergence speed, models trained by the Ours-noH and Ours-noET methods display faster convergence than those trained by the QMIX method. Notably, the Ours-noET algorithm shows the fastest convergence, while Ours-noH converges more slowly, indicating the importance of empirical decomposition for algorithm convergence speed.

Considering the win rate, both the Ours-noH and Ours-noET algorithms outperform the QMIX method, with the Ours-noET algorithm showing a slightly higher win rate. Concerning the battle loss rate, the Ours-noH and Ours-noET methods exhibit slightly lower rates than QMIX, suggesting that both the hierarchical decision network and empirical decomposition contribute similarly to reducing the combat loss rate.

In summary, the improvements introduced by this algorithm generally outperform the QMIX method across convergence speed, stability, the win rate, and the combat loss rate. These experiments validate the effectiveness of the algorithm in addressing multi-UAV combat decision-making problems.

### Disadvantageous combat test

In real air combat, the quantity of enemy UAV is often uncertain, possibly resulting in situations where our side faces a disadvantage. To evaluate the effectiveness of the algorithm proposed in this paper in such scenarios, its performance was tested in various degrees of disadvantageous combat situations (5v8, 6v8, 7v8).

**Figure 5:**
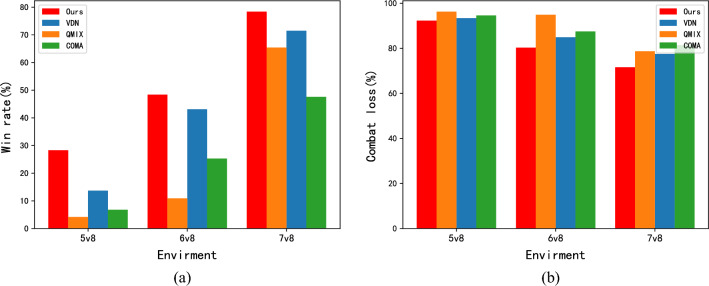
Comparison of disadvantageous combat results. (**a**) The win rate; (**b**) The combat loss rate.

Figure 5 illustrates the results of the algorithm comparison test across various levels of disadvantageous combat environments (5v8, 6v8, 7v8). Our algorithm consistently outperforms others in terms of both the win rate and combat loss rate across all disadvantageous combat tests. Notably, even under the 6v8 disadvantage, our algorithm achieves a win rate close to $50\%$, while the other algorithms fall below this mark. As the disadvantage diminishes, our algorithm exhibits a linear increase in the win rate. Conversely, the QMIX method displays significant fluctuations as the level of disadvantage changes, while our algorithm maintains relatively stable performance. In summary, our method demonstrates the ability to reach a win rate that exceed 50% in disadvantageous confrontations (where the number of fighters is at least 75% of the enemy), highlighting the effectiveness of our algorithm in such scenarios.

### Test with noise

In real air combat environments, the data collected by radar sensors is often affected by noise. If the collected state space data is affected by noise, it will directly affect the accuracy of the UAVs action decision. We test the performance of the proposed algorithm and the baseline algorithm under noise interference. In our tests, we add noise to the enemy missile position, course and speed information to simulate the situation where the radar is subjected to noise interference. The $dp \in [-100m,100m]$ is noise added to enemy missile position data, the $dc \in [-10^{\circ },10^{\circ }]$ is noise added to enemy missile course data and the $ds \in [-20m/s,20m/s]$ is noise added to enemy missile course data. The test results are shown in Table 8.

**Table 8:** Algorithm test results with noise.

Algorithm	Win rate	std	Combat loss	std
Ours(4v4)	**0.534**	**0.052**	**0.765**	**0.031**
QMIX(4v4)	0.492	0.211	0.812	0.075
VDN(4v4)	0.514	0.082	0.839	0.041
COMA(4v4)	0.413	0.068	0.901	0.033
Ours(8v8)	** 0.709**	**0.058**	**0.765**	**0.031**
QMIX(8v8)	0.712	0.093	0.793	0.042
VDN(8v8)	0.601	0.052	0.819	0.049
COMA(8v8)	0.304	0.067	0.841	0.056

Significant values are in bold.

As shown in Table 8, all algorithms show a significant degradation in performance due to the received noise interference, but our algorithm still outperforms the baseline algorithm.

### Results analysis

**Figure 6:**
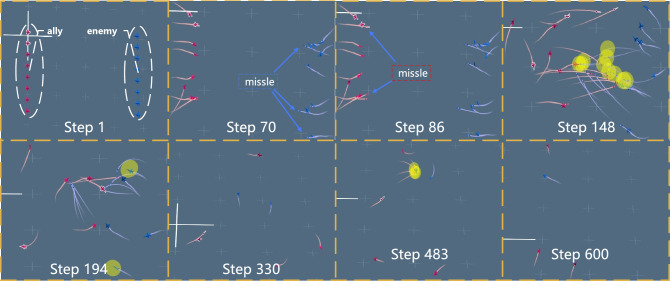
The multi-UAV air combat process (8v8).

Through a comprehensive analysis of the experimental outcomes, we outline the emergent strategies derived from the multi-UAV combat model, as depicted in Fig. 6. In the visual representation, the red side represents allied UAV, the blue side signifies enemy UAV, and the yellow circular area denotes the range of missile explosion damage.

- Efficient attack
  During the initial stages of training, the attack actions of allied UAV controlled by the decision model tend to be random, resulting in frequent ineffective attacks and a depletion of ammunition before the conclusion of the confrontation. As training progresses, a noticeable evolution in behavior emerges. By step 86, allied UAV begin to strategically time their missile launches, opting to deploy them when relatively close to enemy UAV, thereby improving the attack success rates. Conversely, at step 70, enemy UAV choose to launch missiles from maximum range positions.
- Disperse the formation
  At step 148, the allied UAV positioned on the left side of the formation exhibit dispersal after missile launches, aiming to minimize the chances of being targeted by incoming missiles. Conversely, enemy fighters positioned on the right side of the formation are concentrated on the upper right side, significantly increasing their vulnerability to missile attacks. Consequently, the majority of the clustered red fighters shown in step 194 are successfully shot down.
- High-speed circling to find a gaming advantage
  In step 330 depicted in Fig. 6, allied UAV are observed circling rapidly in the upper middle position. This swift movement complicates the ability of the red fighters to find an optimal launching angle (restricted to a maximum of 45 degrees). Simultaneously, our fighters execute high-speed circling maneuvers in the upper right direction, positioning themselves behind the enemy and launching missiles at the most effective angle. Consequently, in step 330, the enemy UAV are successfully shot down. As the round concludes, the number of allied UAV overwhelmingly dominates, securing victory in the game.

## Conclusion

This paper aims to enhance the decision-making efficiency in multi-UAV combat by integrating hierarchical decision-making principles with the experience decomposition and transformation method. It presents a novel multi-UAV combat decision-making approach based on hierarchical reinforcement learning, and the proposed method is evaluated through comparative experiments in 4v4 and 8v8 combat scenarios. Additionally, ablation experiments are designed to analyze the impact of distinct algorithmic enhancements on the performance, behaviors, and strategies of the model. The algorithm performance of the algorithm is further tested in various disadvantageous combat scenarios, and its behavioral strategies are summarized.

The experimental results highlight that the proposed method significantly improves the training speed and decision-making performance of the air combat model. This research provides valuable insights for designing decision-making methods tailored to more complex and realistic multi-UAV combat environments.

While the current focus is on enhancing training efficiency in multi-UAV combat, this study employs a relatively simplified simulation environment. Future research will involve designing more realistic simulation environments, that closely resemble actual air combat scenarios, to validate the algorithmic experiments.

### Supplementary Information

Supplementary Information.

## Data Availability

All data generated or analysed during this study are included in this published article and its supplementary information files.
