# Supplementary material for: Enhancing multi-UAV air combat decision making via hierarchical reinforcement learning
Source: Sci Rep. 2024 Feb 23;14:4458. doi: 10.1038/s41598-024-54938-5 (PMC10891071; doi:10.1038/s41598-024-54938-5)
Supplement: Supplementary file 1 — Supplementary Information. [file 41598_2024_54938_MOESM1_ESM.pdf]

Supplementary materials

Algorithm of hierarchical decision-making multi-aircraft air combat methodology

Algorithm 1 Hierarchical Decision-Making Multi-Aircraft Air Combat Methodology

Require: Air combat combat experience data $o_{f,t}$, $o_{i,t}$, s_t, $a_{f,t}$, $a_{i,t}$
Ensure: Updated network

Initialise $Q_f^n(o_f^n, a_f^n)$, $Q_i^n(o_i^n, a_i^n)$, $Q_f^{\text{tot}}(\tau_f^n, a_f^n)$, $Q_i^{\text{tot}}(\tau_i^n, a_i^n)$, experience buffer $\mathscr{B}$, Number of iterations T, $\varepsilon \in [0,1]$
for $epsoide = 1$ to N **do**
    s_t,$o_{f,t}$,$o_{i,t}$,$a_{f,t}$, $a_{i,t}$, $r_{f,t}$, $r_{i,t}$ = env.reset();
    Initialise the round combat step t_{round};
    for Each step $t = 1$ to T in episode **do**
        if t mod $t_{round} \neq 0$ **then**
            Sampling from the experience buffer was used as input to the network, and using the ε-greedy($Q_f^n(o_{f,t}^n, a_{f,t}^n)$), ε-greedy($Q_i^n(o_{i,t}^n, a_{i,t}^n)$) greedy method to calculate each agent's $a_{f,t}^n$,$a_{a,t}^n$ as (4);
            Calculate s_{t+1}^n, $o_{f,t+1}^n$, $o_{i,t+1}^n$ according to Equation (1) and (2);
            Calculate each agent's $r_{f,t}^n$, $r_{i,t}^n$;
            Feed (s_{t+1}^n, $o_{f,t}^n$, $o_{i,t}^n$, $a_{f,t}^n$, $a_{i,t}^n$, $r_{f,t}^n$, $r_{i,t}^n$,$o_{f,t+1}^n$, $o_{i,t+1}^n$) into π^n;
        else
            Decompose the round combat data π^n into a set of sub-target empirical data π_{sub}^n based on the formula (5);
            Store π_{sub}^n in the experience buffer $\mathscr{B}$;
        end if
        if step t mod $t_{train} = 0$ **then**
            Update $Q_f^n(o_f^n, a_f^n)$, $Q_i^n(o_i^n, a_i^n)$, $Q_f^{\text{tot}}(\tau_f^n, a_f^n)$, $Q_i^{\text{tot}}(\tau_i^n, a_i^n)$ based on Equation (3)
        end if
    end for
end for

$$o_f = \{o^1, ..., o^n\}, \tag{1}$$

$$o_i^m = \omega^m \odot o_f, \tag{2}$$

$$\mathscr{L}(\theta) = \sum_{i=1}^{b} \left[\left(y_i^{\text{tot}} - Q^{\text{tot}}(\tau, a, s; \theta) \right)^2 \right], \tag{3}$$

where, b is the sample batch size for each training, $y^{\text{tot}} = r + \gamma * \max_{\mathrm{a}'} \mathrm{Q}^{\text{tot}} \left(\tau', \mathrm{a}', \mathrm{s}'; \theta^- \right)$, and θ^- stands for the target network parameters . $Q_f^n \left({o_f}^n, {a_f}^n \right)$ and $Q_i^n \left({o_i}^n, {a_i}^n \right)$ represent the action value function of each agent for generating the flying and attacking actions, which are calculated according to Equation (6).

$$a = \begin{cases} \text{argmax}_a Q(o,a), \varepsilon, \\ \text{random action}, 1-\varepsilon, \end{cases} \tag{4}$$

$$\pi^n \mapsto \pi_{\text{sub}}^n : \{\pi_j | d_j = 1, j \in [0, t_{round}]\}, \tag{5}$$

$$Q^\pi(s_t, a_t) = E_{s_{t+1:\infty}, a_{t+1:\infty}}[R_t | s_t, a_t], \tag{6}$$

where, $R_t = \sum_{i=0}^{\infty} \gamma^i r_{t+i}$ represents the discount reward. The strategy learning of each agent in training takes only its own action observation history as input.
